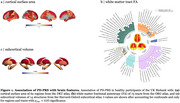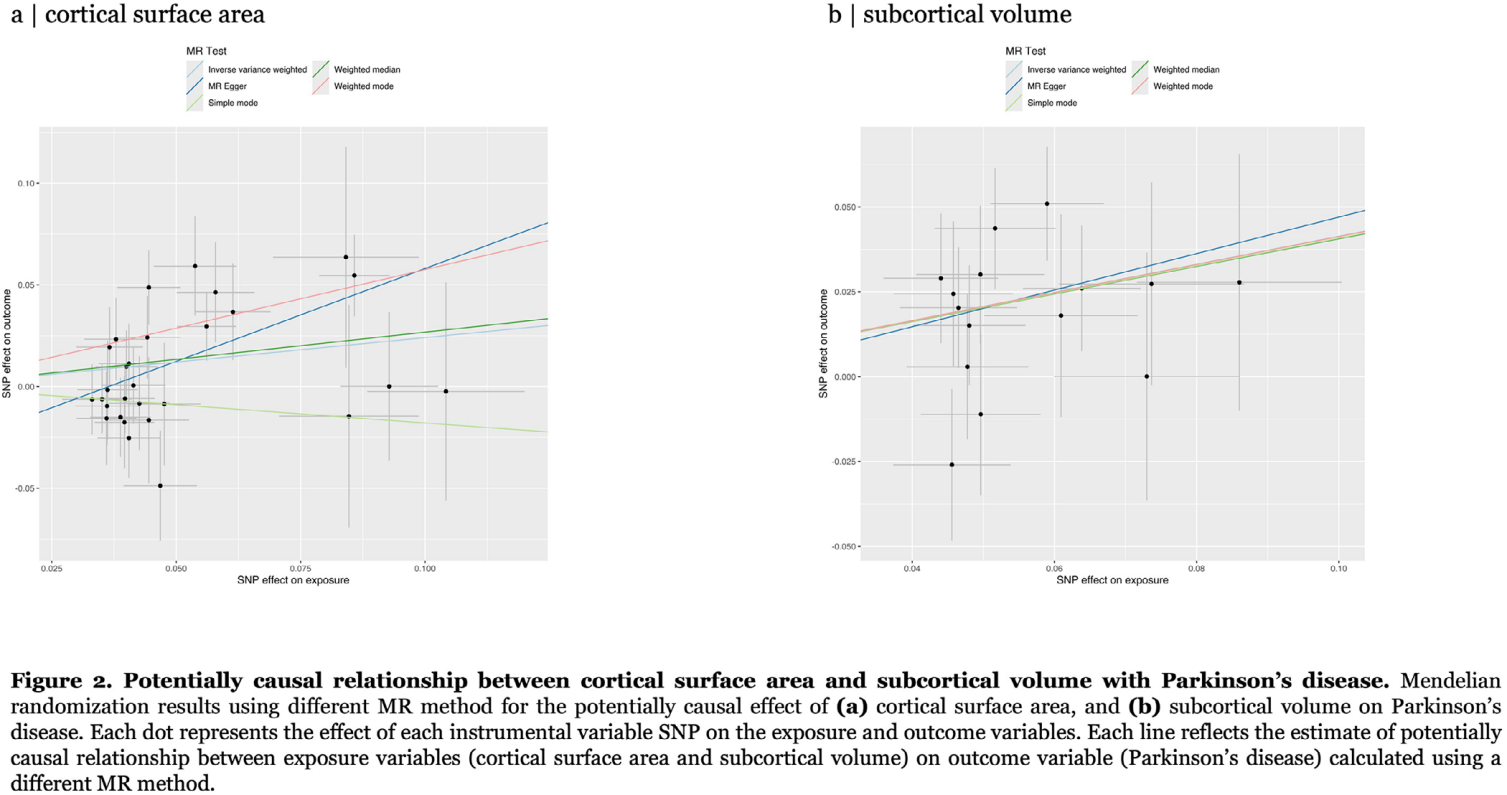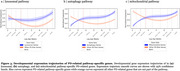# Genetic architecture of Parkinson's disease: investigating the relationships between pathway‐specific polygenic risk scores and neuroanatomical features

**DOI:** 10.1002/alz70856_103914

**Published:** 2025-12-26

**Authors:** Houman Azizi, Alexandre Pastor‐Bernier, Christina Tremblay, Nooshin Abbasi, Lang Liu, Konstantin Senkevich, Moohebat Pourmajidian, Filip Morys, Peter Savadjiev, Eric Yu, Jean‐Baptiste Poline, Ziv Gan‐Or, Yashar Zeighami, Alain Dagher

**Affiliations:** ^1^ McGill University, Montreal, QC, Canada; ^2^ The Neuro (Montreal Neurological Institute), Montreal, QC, Canada; ^3^ Douglas Mental Health University Institute, Montreal, QC, Canada; ^4^ Center of Advanced Research in Sleep Medicine Hôpital du Sacré‐Coeur de Montréal, Montreal, QC, Canada; ^5^ Douglas Mental Health University Institute, Montréal, QC, Canada

## Abstract

**Background:**

Parkinson's disease (PD) is associated with various genetic risk factors and brain structural alterations. However, how these genetic factors influence brain anatomy and potentially contribute to disease risk remains unclear. Here, we aimed to characterize neuroanatomical correlates of PD genetic risk and differentiate between potential genetic factors that affect neurodevelopmental processes and those that contribute to later‐life vulnerability toward PD

**Method:**

Associations between polygenic risk scores of PD (PD‐PRS) and structural and microstructural brain measures were examined using linear regression, and potentially causal relationships between brain structure and PD diagnosis were investigated through Mendelian randomization. Next, PD risk genes were stratified based on their functions into three distinct components of lysosomal, autophagy, and mitochondrial genes, and their pathway‐specific neuroanatomical associations were assessed using linear regression. Finally, we investigated the developmental gene expression trajectories of each pathway using RNA‐sequencing data spanning fetal stages through adulthood and compared them to the expression patterns of other PD risk genes.

**Result:**

PD‐PRS showed widespread positive associations with cortical SA, subcortical volumes, and white matter FA [Figure 1]. Mendelian randomization revealed increased cortical SA and larger subcortical volumes to have a potentially causal effect on PD development [Figure 2]. No significant associations were observed between lysosomal, autophagy, or mitochondrial pathway‐specific PD‐PRSs and brain structural measures. Developmental gene expression trajectory analyses revealed distinct patterns of expression for mitochondrial and autophagy pathway genes, showing significantly lower expression during fetal stages compared to other PD risk genes [Figure 3].

**Conclusion:**

Our findings reveal a link between PD genetic risk and brain structure, indicative of greater size of grey matter and higher white matter integrity, potentially leading to increased risk of PD development. Additionally, the lower expression of mitochondrial and autophagy pathway genes during fetal stages as well as the non‐significant associations between pathway‐specific polygenic risk scores and neuroanatomical features suggest that these pathways may contribute to disease risk through mechanisms independent of early neurodevelopmental processes. These results provide new insights into how genetic risk factors might shape brain structure and contribute to PD susceptibility, highlighting the complex interplay between developmental and pathway‐specific mechanisms in PD pathogenesis.